# Dual Lipolytic Control of Body Fat Storage and Mobilization in *Drosophila*

**DOI:** 10.1371/journal.pbio.0050137

**Published:** 2007-05-08

**Authors:** Sebastian Grönke, Günter Müller, Jochen Hirsch, Sonja Fellert, Alexandra Andreou, Tobias Haase, Herbert Jäckle, Ronald P Kühnlein

**Affiliations:** 1 Abteilung Molekulare Entwicklungsbiologie, Max-Planck-Institut für Biophysikalische Chemie, Göttingen, Germany; 2 Therapeutic Department Metabolic Diseases, Sanofi-Aventis Pharma Deutschland GmbH, Frankfurt, Germany; University of Cambridge, United Kingdom

## Abstract

Energy homeostasis is a fundamental property of animal life, providing a genetically fixed balance between fat storage and mobilization. The importance of body fat regulation is emphasized by dysfunctions resulting in obesity and lipodystrophy in humans. Packaging of storage fat in intracellular lipid droplets, and the various molecules and mechanisms guiding storage-fat mobilization, are conserved between mammals and insects. We generated a *Drosophila* mutant lacking the receptor (AKHR) of the adipokinetic hormone signaling pathway, an insect lipolytic pathway related to ß-adrenergic signaling in mammals. Combined genetic, physiological, and biochemical analyses provide in vivo evidence that AKHR is as important for chronic accumulation and acute mobilization of storage fat as is the Brummer lipase, the homolog of mammalian adipose triglyceride lipase (ATGL). Simultaneous loss of Brummer and AKHR causes extreme obesity and blocks acute storage-fat mobilization in flies. Our data demonstrate that storage-fat mobilization in the fly is coordinated by two lipocatabolic systems, which are essential to adjust normal body fat content and ensure lifelong fat-storage homeostasis.

## Author Summary

The amount of body fat that an animal stores is a critical parameter for its survival. Although under-storage of fat creates risk during periods of famine, over-storage also impairs fitness—obesity in humans is associated with severe health threats, such as cardiovascular disease, type II diabetes, and cancer. A delicate balance between two antagonistic processes adjusts body fat storage: lipogenesis produces fat stores, and lipolysis mobilizes fat. It is unclear, however, how many regulatory systems orchestrate lipolysis in animals, whether these systems are evolutionarily conserved, and to what extent impaired lipolytic regulation contributes to excessive body fat accumulation. We show that in the fruit fly *Drosophila,* lipolysis is under dual control. Inactivation of either of the two control pathways generates flies with excessive fat accumulation and limited fat-mobilization capability. Mutant flies simultaneously lacking key genes of both lipolytic systems, however, are extremely obese and completely blocked in body fat mobilization even when fully food deprived. Interestingly, our study reveals that key components and regulatory mechanisms of lipolysis are evolutionarily conserved between insects and mammals, making the fruit fly a valuable model system for research on lipid metabolism.

## Abstract 3

Simultaneous loss of the receptor for adipokinetic hormone and the Brummer triglyceride lipase causes extreme obesity and blocks acute storage fat mobilization in flies.

## Introduction

Tightly regulated storage-fat accumulation and mobilization are a central characteristic of organismal energy homeostasis. In organisms as different as flies and man, body fat reserves are primarily stored as triacylglycerol (TAG) in lipid droplets [1,2], which are intracellular organelles most prominent in specialized storage tissues such as insect fat body or mammalian adipose tissue. A finely tuned balance between lipid synthesis (lipogenesis) and lipid mobilization (lipolysis) adjusts the fat storage level within cells.

In humans, the disruption of this balance is linked to complex metabolic disorders such as obesity and type II diabetes, and is causative for monogenetic neutral lipid storage diseases (NLSD) such as Chanarin-Dorfman Syndrome (CDS) [3,4]. The accumulation of TAG-containing lipid droplets in multiple tissues characteristic of CDS has been linked to impaired lipolysis caused by mutations in *comparative gene identification 58 (CGI-58;* also called *ABHD5)* [5]. CGI-58 acts as a coactivator of adipose triglyceride lipase (ATGL [6], also called TTS, desnutrin [7], calcium-independent phospholipase A2 [8], or patatin-like phospholipase domain-containing protein 2) [9]. Recently, it was shown that patients carrying ATGL gene mutations suffer from increased systemic TAG accumulation (so called NLSD with myopathy, [10]), supporting the idea that the impaired activation of ATGL contributes to the pathogenesis of CDS. The findings that polymorphisms in human ATGL are associated with plasma levels of TAG and free fatty acids (FFA) [11] and that ATGL knockout mice are obese [12] further underscores the central role of ATGL in mammalian lipolysis.

ATGL is ubiquitously expressed; however, it is strongly enriched in adipose tissue, where it acts in concert with hormone-sensitive lipase (Hsl) to execute lipolysis at the lipid droplet surface (for review, see [13,14]). Initiation of lipid mobilization is controlled by lipolytic hormones that act via ß-adrenergic receptor signaling. According to the current model, ß-adrenergic receptor stimulation activates protein kinase A (PKA), which subsequently phosphorylates Hsl and the lipid droplet scaffold protein perilipin. PKA activation promotes the translocation of cytoplasmic Hsl to the lipid droplet surface in a manner dependent on the phosphorylation state of perilipin (for review, see [15]). ATGL activity is indirectly activated by PKA signaling via the phosphorylation-triggered release of its perilipin-bound activator CGI-58 [16,17]. Notably, starvation-induced increase of ATGL transcript levels is dependent on glucocorticoid, but not on cyclic adenosine monophosphate (cAMP)/PKA signaling [7], suggesting that ATGL activity is controlled by various regulatory inputs.

ATGL function is highly conserved during evolution; ATGL-related proteins have been identified as key regulators of yeast, plant, and insect lipometabolism (Saccharomyces cerevisiae Tgl4 [18,19], Arabidopsis thaliana SUGAR-DEPENDENT1 [20], and Drosophila melanogaster Brummer [21]).

Despite their anatomical and physiological differences, there is remarkable evolutionary conservation of lipolytic factors and mechanisms between mammals and insects. Like the ATGL knockout mice, *brummer (bmm)* mutant flies are obese and impaired in acute lipid mobilization [12,21]. Comparable with ß-adrenergic signaling in mammalian adipose tissue, initiation of storage-fat mobilization in the insect fat body relies on hormonal signaling via the adipokinetic hormone (AKH) pathway (for review, see [22,23]). Starvation-induced release of AKH from neuroendocrine corpora cardiaca cells of the ring gland triggers signaling by G protein–coupled AKH receptor (AKHR) [24], activates PKA, and controls both *Drosophila* hemolymph sugar homeostasis [25,26] and larval lipolysis [25,27]. The primary target of AKH-dependent PKA phosphorylation in the tobacco hornworm Manduca sexta is the perilipin homolog LSDP-1 (a synonym for lipid storage droplet-1 [LSD-1]) [28]. Activation of lipid droplets by phosphorylation of LSDP-1 mediates most of the AKH-induced lipolysis in *Manduca* [29]. In *Drosophila,* another perilipin relative called LSD-2 has been demonstrated to be crucial for fat-storage regulation [30,31]. It is currently unknown what the identity of the TAG lipase(s) executing the AKH-induced fat-mobilization program in the fly is. Recently, *Drosophila* CG8552, the homolog of the Manduca sexta TG-lipase, has been proposed to implement AKH-dependent lipolysis [32], but its in vivo role has still to be analyzed. Remarkably, starvation also stimulates lipid mobilization by an uncharacterized, AKH-independent mechanism in adult *Manduca* [33], suggesting that, like in mammals, insect lipolysis is under control of multiple regulatory systems.

To address the question of how many lipocatabolic systems orchestrate acute lipolysis in response to energy shortage in animals and to what extent chronic dysregulation of inducible lipolytic systems contributes to fat-storage diseases, we analyzed the function of the *Drosophila* AKHR in vivo. Here we show that AKHR mutant flies become obese and are impaired in storage-fat mobilization. Flies lacking AKHR and Brummer lipase activity demonstrate that acute storage-fat mobilization in *Drosophila* is coordinated by two regulatory systems, which may communicate in a compensatory manner to ensure lifelong fat-storage homeostasis.

## Results/Discussion

**Figure 1:**
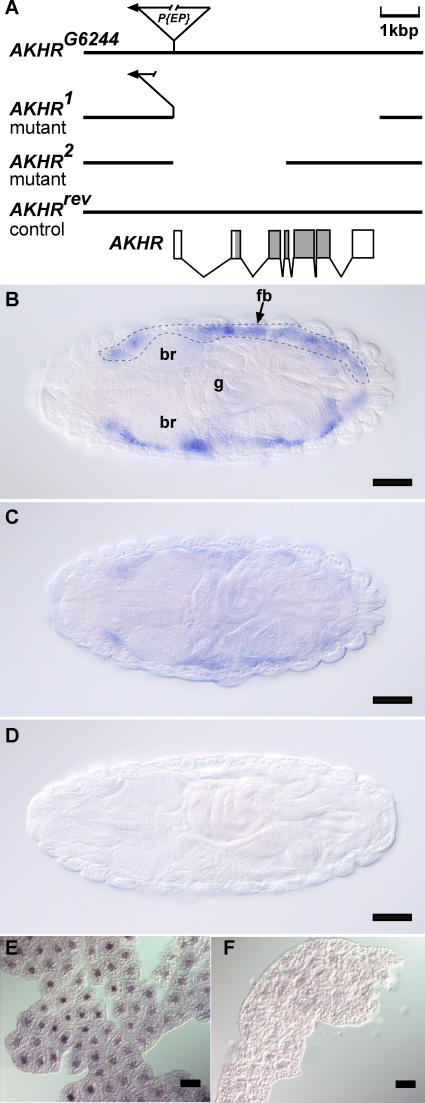
Molecular Organization, Mutants, and Fat Body Expression of the *AKHR* Gene (A) Genomic organization of the *AKHR* gene represented by the *AKHR* cDNA comprised of seven exons (grey boxes: coding exons; open boxes: UTRs). *AKHR^G6244^* flies carrying a *P{w^+mC^ = EP}* insertion in the first *AKHR* exon were used to generate *AKHR* deletion mutants *(AKHR^1^, AKHR^2^)* and genetically matched control flies *(AKHR^rev^)* having an intact *AKHR* gene. (B–F) In situ hybridization showing expression of the fat body marker gene *Adh* (B) and *AKHR* expression in fat body tissue during late embryonic (C) and third instar larval stages (E) lacking in *AKHR^1^* mutants ([D] and[F]). All embryos are depicted in dorsal view, anterior is left. Scale bar represents 50 μm. br, brain; fb, fat body; g, gut.

Expression studies in a heterologous tissue culture system [24] and in *Xenopus* oocytes [34] identified AKH-responsive G protein–coupled receptors in *Drosophila,* such as the one encoded by the *AKHR* (or *CG11325*) gene (FlyBase name: *Gonadotropin-releasing hormone receptor [GRHR]*). *AKHR* is expressed during all ontogenetic stages of the fly ([35] and unpublished data). It consists of seven exons, which encode a predicted protein of 443 amino acids (Figure 1A and [36]). In late embryonic and larval stages, *AKHR* is expressed in the fat body (Figure 1C and 1E; compare this to the expression pattern of the fat body marker gene *Alcohol dehydrogenase [Adh]* in Figure 1B). This finding is consistent with its predicted role as transmitter of the lipolytic AKH signal in this organ.

In order to examine the effect of *AKHR* signaling on fat storage and mobilization in vivo, two different P element–insertion mutants were used, *CG11188^A1332^* and *AKHR^G6244^,* which are located close to and within the *AKHR* gene, respectively. *CG11188^A1332^* flies carrying the transposable element integration designated A1332 allow for the transcriptional activation of the adjacent *AKHR* gene (Figure S1A and unpublished data). This ability was used for *AKHR* gain-of-function studies by overexpression of *AKHR* in the fat body of flies. As shown in Figure S1B, overexpression of AKHR in response to a fat body–specific *Gal4* inducer causes dramatic reduction of organismal fat storage. This finding could be recapitulated by fat body–targeted *AKHR* expression from a cDNA-based upstream activation sequence (UAS)-driven *AKHR* transgene (Figure S1B). These gain-of-function results suggest a critical in vivo role for *AKHR* in storage-lipid homeostasis of the adult fly.

**Figure 2:**
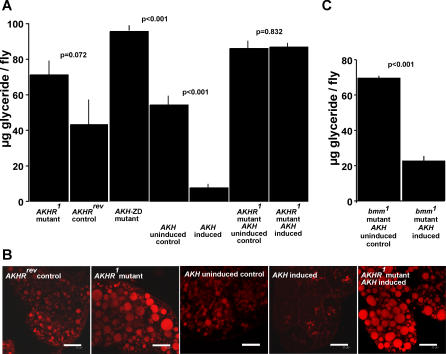
AKH-Dependent Storage Fat Mobilization Strictly Depends on *AKHR,* but Not on *brummer* Lipase Function (A and B) Organismal fat content (A) and cellular phenotype of fat-storage tissue visualized by Nile red staining of lipid storage droplets (B) show excessive fat storage in *AKHR^1^* mutants and in flies lacking AKH-positive neuroendocrine cells by *reaper*-induced apoptosis (*AKH*-ZD mutants; for details see Materials and Methods) compared to the *AKHR^rev^* control. AKH-dependent depletion of fat storage (compare *AKH* induced vs. *AKH* uninduced control in [A] and [B]) is blocked in flies lacking AKHR function (compare *AKHR^1^* mutant *AKH* induced vs. *AKHR^1^* mutant *AKH* uninduced control in [A] and [B]). Scale bar represents 25 μm. (C) AKH induction reduces fat storage in *bmm* mutants (compare *bmm^1^ AKH* induced vs. *bmm^1^ AKH* uninduced control).

**Figure 3:**
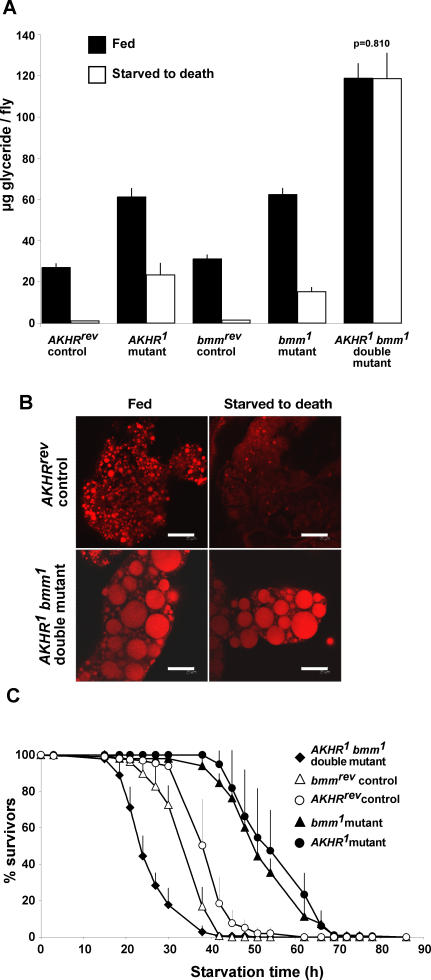
Severe Obesity and Impaired Lipid Mobilization in *AKHR brummer* Double-Mutant Flies (A and B) Organismal fat content (A) and Nile red staining of lipid storage droplets in fat body tissue (B) demonstrate extreme obesity of ad libitum–fed *AKHR^1^ bmm^1^* double mutants (glyceride content doubled compared to *AKHR^1^* or *bmm^1^* single mutants, quadrupled compared to genetically matched controls *[AKHR^rev^* or *bmm^rev^]* having wild-type *AKHR* and *bmm* function; filled bars). Induced storage-fat mobilization in response to starvation is impaired in *AKHR^1^* and *bmm^1^* single mutants, but blocked in *AKHR^1^ bmm^1^* double mutants (open bars in [A]). (C) Survival curves demonstrate starvation resistance of obese *AKHR^1^* and *bmm^1^* single mutants, but starvation sensitivity of extremely obese *AKHR^1^ bmm^1^* mutants compared to genetically matched controls *(AKHR^rev^* or *bmm^rev^)*. Scale bar represents 25 μm. Note: Except where given, *p* is less than 0.001 for all comparisons between mutant and control, and fed versus starved to death conditions.

Flies of strain *AKHR^G6244^,* which carry a P element integration in the *AKHR* untranslated leader region, were used to generate the *AKHR* deletion mutants *AKHR^1^* and *AKHR^2^,* as well as the genetically matched control *AKHR^rev^,* which possesses a functionally restored *AKHR* allele (Figure 1A). As exemplified for embryonic and larval stages (Figure 1D and 1F), *AKHR^1^* mutants lack *AKHR* transcript. Ad libitum–fed flies without *AKHR* function are viable, fertile, and have a normal lifespan (unpublished data). However, such flies accumulate lipid storage droplets in the fat body and have 65%–127% more body fat than the controls (Figures 2A, 2B, and 3A). These results indicate that *AKHR^1^* mutants develop an obese phenotype. The same result was obtained with *AKHR^2^* and *AKHR^1^/AKHR^2^* transheterozygous mutant flies (unpublished data), as well as with flies lacking the AKH-producing cells of the neuroendocrine system due to targeted ablation by the cell-directed activity of the proapoptotic gene *reaper* (*AKH-*ZD mutant in Figure 2A). Conversely, chronic overexpression of AKH provided by a fat body–targeted AKH transgene of otherwise wild-type flies largely depletes lipid storage droplets and organismal fat stores (Figure 2A and 2B). However, the obese phenotype of *AKHR* mutants is unresponsive to AKH (Figure 2A and 2B), indicating that *AKHR* is the only receptor transmitting the lipolytic signal induced by AKH in vivo. Collectively, these data demonstrate that AKH-dependent *AKHR* signaling is critical for the chronic lipid-storage homeostasis in ad libitum–fed flies.

Studies on various insect species helped elucidate several components and mediators of the lipolytic AKH/AKHR signal transduction pathway (for review, see [22]). However, the identity of the TAG lipase(s) executing the AKH-induced fat mobilization program remained elusive. Besides the *Drosophila* homolog of the TG lipase from the tobacco hornworm Manduca sexta [32], the recently identified Brummer lipase, a homolog of the mammalian ATGL, is a candidate member of the *AKH/AKHR* pathway. This is based on the striking similarity between the phenotypes of *AKHR* and *bmm* mutants. Ad libitum–fed flies lacking either *AKHR* or *bmm* activity, store excessive fat (Figures 2A and 3A; and [21]), predominantly as TAG (Figure S3). Both mutants show incomplete storage-fat mobilization (Figure 3A and [21]) and starvation resistance (Figure 3C and [21]) in response to food deprivation. Starvation resistance of these mutants might be caused by their increased metabolically accessible fat stores (Figure 3) and/or changes in their energy expenditure due to locomotor activity reduction as described for flies with impaired AKH signaling [25,27]. Despite the phenotypic similarities of their mutants, however, *AKHR* and *bmm* are members of two different fat-mobilization systems in vivo. Several lines of evidence support this conclusion. On one hand, AKH overexpression reduces the excessive TAG storage of *bmm* mutants (Figure 2C), while on the other, *bmm*-induced fat mobilization can be executed in *AKHR* mutants (Figure S2). Thus, AKH/AKHR signaling is not a prerequisite for Brummer activity. Moreover, genetic epistasis experiments support this idea that *AKHR* and *bmm* belong to different control systems of lipocatabolism in vivo. Double-mutant analysis reveals that the obesity of *AKHR* and *bmm* single mutants is additive. Accordingly, *AKHR bmm* double-mutant flies store about four times as much body fat as control flies and accumulate excessive lipid droplets in their fat body cells (Figure 3A and 3B).

Thin layer chromatography (TLC) analysis was used to compare the storage-fat composition of *AKHR* and *bmm* single mutants with *AKHR bmm* double-mutant and control flies (Figure S3). Excessive body fat accumulation in *AKHR bmm* double mutants is on the one hand due to TAG, which is increased compared to *AKHR* and *bmm* single-mutant flies. Additionally, an uncharacterized class of TAG (TAGX; for details see Protocol S1) appears exclusively in *AKHR bmm* double mutants (Figure S3 and unpublished data). In contrast to TAG, changes in diacylglycerol (DAG) content do not substantially contribute to the differences in body fat content in any of the analyzed genotypes (Figure S3). Taken together a quantitative increase and a qualitative change in the TAG composition account for the extreme obesity in *AKHR bmm* double-mutant flies.

To address the in vivo response of *AKHR bmm* double mutants to induced energy-storage mobilization, flies were starved and their survival curve monitored. *AKHR bmm* double mutants die rapidly after food deprivation (Figure 3C). In contrast to the starvation-resistant obese *AKHR* and *bmm* single mutants, the double mutants are not capable of mobilizing even part of their excessive fat stores (Figure 3A). *AKHR bmm* double mutants do not, however, suffer from a general block of energy-storage mobilization because they can access and deplete their carbohydrate stores (Figure S4). These data demonstrate that energy homeostasis in *AKHR bmm* double-mutant flies is imbalanced by a severe and specific lipometabolism defect, which cannot be compensated in vivo.

The nature of Brummer as a TAG lipase and AKHR as a transmitter of lipolytic AKH signaling suggests that the extreme storage-fat accumulation and starvation sensitivity of ad libitum–fed *AKHR bmm* double mutants is due to severe lipolysis dysfunction. To address this possibility in vitro, lipolysis rate measurements on fly fat body cell lysates and lysate fractions of control flies were performed. Results, summarized in Figure S5A, show that the cytosolic fraction of fat body cells contains the majority of basal and starvation-induced lipolytic activity against TAG, similar to the activity distribution in mammalian adipose tissue [37]. Little basal and induced total TAG cleavage activity localizes to the lipid droplet fraction, whereas the pellet fraction including cellular membranes shows low basal, non-inducible TAG lipolysis. Lipolysis activity against DAG is similarly distributed between fat body cell fractions (Figure S5B). However, in accordance with the function of DAG as major transport lipid in *Drosophila* [38], DAG lipolysis in fat body cells is not induced in response to starvation (Figure S5B).

**Figure 4:**
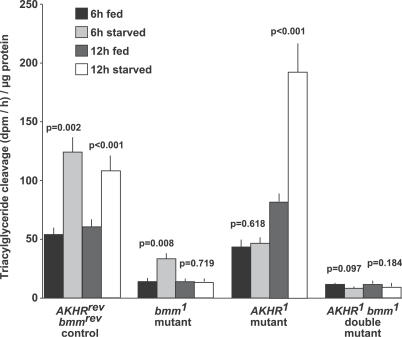
Impaired Basal and Blocked Starvation-Induced TAG Lipolysis in Fat Body Cells Lacking Both *AKHR* and *brummer* Gene Function Fat body cells of control flies *(AKHR^rev^ bmm^rev^)* exhibit basal TAG lipolysis, which is doubled by starvation-induced lipolysis after 6 h or 12 h of food deprivation. *bmm* mutant cells have reduced basal lipolysis and lack induced lipolysis after 12 h starvation. *AKHR* mutant cells lack early (6 h) induced lipolysis, but show strong starvation-induced lipolysis after 12 h food deprivation. *AKHR bmm* double mutants have reduced basal lipolysis and lack starvation-induced lipolysis altogether.

Based on the lysate fraction analysis of control flies, cytosolic fat body cell extracts were used to assess the basal and starvation-induced lipolytic activity of mutant and control flies on TAG, DAG, and cholesterol oleate substrates. Whereas DAG and cholesterol oleate cleavage activity of fat body cells is comparable between all genotypes and physiological conditions tested (Figure S5C and S5D), TAG lipolysis varies widely (Figure 4). Compared to control flies, basal TAG lipolysis of *AKHR bmm* double mutants is reduced by 80% and induced TAG cleavage is completely blocked, consistent with the flies' extreme obesity and their inability to mobilize storage fat. The impairment of basal lipolysis in the double mutants is largely due to the absence of *bmm* function, because it is also detectable in *bmm* single-mutant cells, whereas basal lipolysis in *AKHR* mutants is not reduced. Interestingly, *bmm* mutants mount a starvation-induced TAG lipolysis response after short-term (6 h), but not after extended (12 h), food deprivation. Conversely, *AKHR* mutant cells lack an early lipolysis response, but exhibit strong TAG cleavage activity after extended food deprivation. These data suggest that induced storage-fat mobilization in fly adipocytes relies on at least two lipolytic phases: an early, AKH/AKHR-dependent phase and a later, Brummer-dependent phase. Accordingly, we speculate that the obesity of *bmm* and *AKHR* mutant flies is caused by different mechanisms: chronically low basal lipolysis in *bmm* mutants and, in *AKHR* mutants, lack of induced lipolysis during short-term starvation periods that is characteristic of organisms with discontinuous feeding behavior. We acknowledge, however, that in vitro lipolysis assays on artificially emulsified substrates allow only a limited representation of the lipocatabolism in vivo, because lipid droplet–associated proteins modulate the lipolytic response in the insect fat body [28,29] and mammalian tissue [9,15,16]. Moreover, excessive fat accumulation in *AKHR* mutants may be in part due to increased lipogenesis because AKH signaling has been demonstrated to repress this process in various insects [39–41].

**Figure 5:**
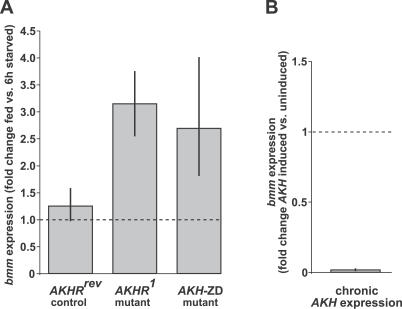
Antagonistic Transcriptional Regulation of *brummer* Lipase in Response to AKH/AKHR Lipolytic Signaling (A) Moderate transcriptional up-regulation of *bmm* in control flies *(AKHR^rev^)* after 6 h food deprivation, but starvation-induced hyperstimulation of *bmm* transcription in obese *AKHR* mutants *(AKHR^1^)* and flies lacking the AKH-producing neuroendocrine cells (*AKH*-ZD). By contrast, *bmm* transcription in lean flies chronically expressing *AKH* in the fat body (B) is strongly reduced.

The finding of the dual lipolytic control in the fly raises the question of whether the two systems involved act independently of each other or whether one system responds to the impairment of the other. Modulation of transcription is an evolutionarily conserved regulatory mechanism of lipases from the ATGL/Brummer family. ATGL is transcriptionally up-regulated in fasting mice [7], as is *bmm* transcription in starving flies ([21] and Figure 5A). Moreover *bmm* overexpression depletes lipid stores in the fat body of transgenic flies ([21] and Figure S2). Accordingly, we analyzed *bmm* transcription in response to modulation of AKH/AKHR signaling to assess a potential regulatory interaction between the two lipolytic systems. Compared to the moderate starvation-induced up-regulation of *bmm* in control flies, the gene is hyperstimulated in flies with impaired AKH signaling. As early as 6 h after food deprivation, *bmm* transcription is up-regulated by a factor of 2.5–3 in flies lacking the AKH-producing neuroendocrine cells (*AKH*-ZD) or in *AKHR* mutant flies (Figure 5A). Conversely, chronic expression of AKH in the fat body suppresses *bmm* transcription (Figure 5B). *Bmm* hyperstimulation in *AKHR* mutants is consistent with a subsequent strong increase of starvation-induced TAG lipolysis observed 12 h after food deprivation (Figure 4). Taken together, these data demonstrate an AKH/AKHR-independent activation mechanism of *bmm* and suggest the existence of compensatory regulation between *bmm* and the AKH/AKHR lipolytic systems, the mechanism of which is currently unknown.

The results presented here provide in vivo evidence that the fly contains two induced lipolytic systems. One system confers AKH/AKHR-dependent lipolysis, a signaling pathway, which assures rapid fat mobilization by cAMP signaling and PKA activity. *Drosophila*'s second lipolytic system involves the Brummer lipase, which is responsible for most of the basal and part of the induced lipolysis in fly fat body cells, likely via transcriptional regulation. Currently, it is unknown whether Brummer activity is post-translationally modulated by an α/ß hydrolase domain-containing protein like the regulation of its mammalian homolog ATGL by CGI-58. Homology searches between mammalian and *Drosophila* genomes identify the *CGI-58*–related fly gene *CG1882* and the putative Hsl homolog CG11055, providing additional support for the evolutionary conservation of fat-mobilization systems. However, differences in lipid transport physiology (i.e., DAG transport in *Drosophila,* and FFA in mammals) suggest a different substrate specificity or tissue distribution of fly Hsl compared to its mammalian relative.

Future studies will not only unravel the crosstalk between the two *Drosophila* lipocatabolic systems, but also disclose the identity of additional genes involved in this process, such as the upstream regulators of *bmm*. Our study substantiates the emerging picture of the evolutionary conservation between insect and mammalian fat-storage regulation and emphasizes the value of *Drosophila* as a powerful model system for the study of human lipometabolic disorders.

## Materials and Methods

### Fly techniques.

Fly strains used in this study are summarized in Table 1. Flies were propagated as described [21]. Flies of the genotype w^*^ ; P{w^+mC^ = EP}AKHR^G6244^ (previously called w^*^ ; P{w^+mC^ = EP}AKHR^GE16070^) were obtained from GenExel, (http://genexel.com). The EP transposon construct [42] integration in this fly stock was localized by sequencing to chromosome 2L between positions 6716140 and 6716139 (FlyBase D. melanogaster Genome Release 4.2.1) corresponding to positions 44/45 in the 5′ untranslated leader region of *AKHR* (details are available on request). *AKHR^1^* and *AKHR^2^* deletion mutants missing genomic DNA sequences 2L 6711184 to 6716139 and 2L 6713381 to 6716139, respectively, corresponding to positions −1448 to +3507 and −1448 to +1310 relative to putative AKHR start ATG, causing lack of the complete *AKHR* coding region *(AKHR^1^)* and of AKHR amino acid positions 1–165 *(AKHR^2^),* as well as flies carrying the precise excision alleles *AKHR^revA^* and *AKHR^revB^* (collectively called *AKHR^rev^*), were generated by a conventional P element–mobilization scheme and molecularly characterized by sequencing the relevant part of the *AKHR* gene (Note: *AKHR^1^* contains 289 base-pair [bp] and *AKHR^2^* 18-bp residual P element sequences). Because *AKHR^revA^* and *AKHR^revB^* behaved indistinguishably in the assays tested, *AKHR^revA^* was used in all experiments labeled with *AKHR^rev^,* with the exception of Figure 1C *(AKHR^revB^)* and Figure 3C *(AKHR^revA^/AKHR^revB^).*

**Table 1:**
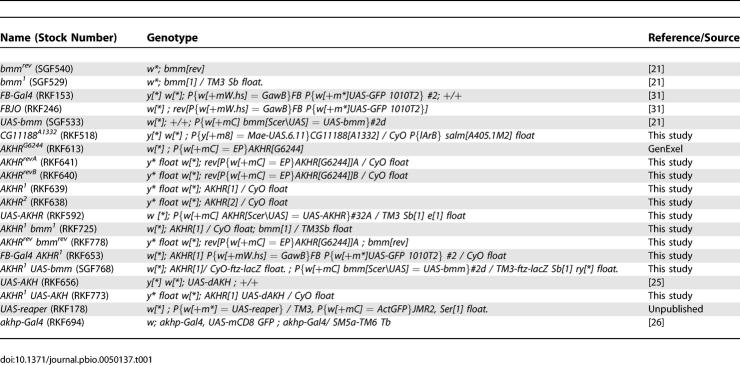
Names, Genotypes, and References of Fly Stocks

The fly strain *CG11188^A1332^* was recovered as an autosomal integration line in a X-chromosomal gain-of-function screen using the *P{Mae-UAS.6.11}* transposable element [43]. The A1332 integration site was determined as described [43] and localized at chromosome 2L between positions 6717708/9 (FlyBase D. melanogaster Genome Release 4.2.1) corresponding to positions 82/83 in the 5′ untranslated leader region of *CG11188-RA*.

To generate transgenic fly strains allowing conditional expression of *AKHR* (UAS-*AKHR*), *AKHR* cDNA GH19447 was cloned into vector pUAST (see below). Transgenic fly stocks were established by P element–mediated germline transformation as described [31]. *AKH-*ZD flies lacking the AKH-positive neuroendocrine cells of the corpora cardiaca [25,26] were generated by crossing *akhp-Gal4* flies to *UAS-reaper* flies.

### Physiology.

Organismal fat (expressed as glycerides) and glycogen content of 6-d-old male flies were quantified as described [21,31]. Prior to glycogen measurements, fed flies were food deprived for 2 h to reduce recently ingested carbohydrates in the digestive tract. Post mortem residual fat and glycogen content was determined using flies subjected to starvation and collected 0–12 h after death. Depicted are representative experiments with average values of triplicate measurements (except in Figure 3A in which ten replicates are shown) and corresponding standard deviations. Experiments were repeated at least twice.

For starvation assays, 5–10 × 20 male flies of each genotype, 6–7 d of age, were transferred to vials provided with water supply only. Mortality rates were determined by regularly counting the number of dead flies as diagnosed by the lack of sit-up response. Plotted are average survival-rate values and the corresponding standard deviations of a representative experiment.

### Lipolysis assays.

Abdominal adipocytes from 100–200 immature adult male flies (0–6 h of age) and subsequently fed or starved for 6 h or 12 h were manually released as described [21] and snap frozen in a minimal volume of PBS. The adipocytes of each genotype were resuspended in 22.5 μl buffer A (25 mM Tris/HCl [pH 7.4], 250 mM sucrose, 5 mM NaF, 10 mM NaPP_i_, 1 mM Na_3_VO_4_, 1 mM EDTA, 20 μg/ml leupeptin, 1 mM benzamidine, 0.5 mM PMSF), and protein content was determined using the BCA method (Pierce Biotechnology, http://www.piercenet.com). The adipocytes were homogenized in buffer A at approximately 1 mg/ml protein concentration. After centrifugation (1,000×*g,* 5 min, 15 °C), the supernatant was used as total fly-adipocyte lysate. For separation of fly-adipocyte lipid droplets (LD) and cytosol, 0.5 ml of the total lysate was combined with 2.5 ml of 65% sucrose (w/v), and poured into a 5-ml centrifuge tube. A total of 1.5 ml of 10% sucrose (w/v) was then layered on top of the sucrose cushion. The tube was filled to capacity with buffer A. The gradient was centrifuged (172,000×*g,* 60 min, 15 °C) and then allowed to coast to rest. The most buoyant white layer of the gradient containing the floating LD was isolated by suction with a syringe (1 ml). About 2.5 ml of the 65% sucrose cushion were recovered and used as cytosol. LD were recovered from the surface layer after a washing cycle with 10 ml of buffer A (20,000×*g,* 10 min) and their volume adjusted to 1 ml with buffer A. The pellet obtained by the gradient centrifugation and containing total fly-adipocyte membranes was suspended in 100 μl of buffer A. Cytosolic extracts were used for assaying lipolysis of the different genotypes because these extracts account for the majority of the lipolytic activity recovered with total fly-adipocyte lysates (Figure S5A and S5B).

TAG cleavage of 3–10 μl total lysates, LD, membranes (Figure S5A), and cytosol (Figures 4 and S5A) was measured by incubation with [^3^H]-trioleoylglyceride (emulsified with phosphatidylcholine and phosphatidylinositol by ultrasonic treatment) in buffer C (50 mM Tris/HCl [pH 7.4], 1 mM EDTA, 500 mM NaCl, 5 mM KCl, 1 mM MgCl_2_, 2% BSA, 1 mM DTT, 1 mM benzamidine, 5 μg/ml leupeptin, 20 μg/ml aprotinin) in a total volume of 50 μl. After alkaline chloroform/methanol partitioning, the amount of [^3^H]oleate liberated was determined and used for calculation of triglyceride cleavage activity as described previously [44] with modifications [45].

### Molecular and cell biology.

The *AKHR* cDNA clone pOT2 GH19447 [46] (RK228) was received from Invitrogen (http://www.invitrogen.com) and its insert confirmed by full-length sequencing to correspond to *CG11325-RA* [36]. To generate UAS-*AKHR,* GH19447 sequences corresponding to *CG11325-RA* positions 181 to 2,032 were PCR-amplified and subcloned into pUAST [47] to generate pUAST-*AKHR* (RK230) (details are available on request).

In situ hybridization on whole-mount embryos and third instar larval tissue using a digoxigenin-labeled antisense RNA probe was performed as described [31]. A 1.3-kilobase *AKHR* antisense probe was generated by in vitro transcription using SP6 RNA-Polymerase on pOT2 GH19447 template linearized with EcoRV. Dr. Gerd Vorbrüggen generously provided an antisense probe against the *Adh* gene, which was generated by in vitro transcription using SP6 RNA-Polymerase on pOT2 GH01616 [46] template linearized with EcoRI.

For quantitative reverse-transcriptase PCR (Q-RT-PCR), total RNA was prepared from flies of the respective genotypes using the peqGold TriFast reagent (peqlab; http://www.peqlab.de) and reverse transcribed using the SuperScript choice cDNA synthesis system (Invitrogen). The Q-RT-PCR reactions were performed on an Eppendorf Mastercycler ep realplex using Qiagen (http://www1.qiagen.com) QuantiTect primers QT00964460 and QT00950474 to quantify *bmm* and—for normalization—*ß-Tub56D* transcripts, respectively. Samples were analyzed in triplicate, and experiments were repeated at least twice. Fold regulation and the regulation range (black bars in Figure 5) were calculated using the comparative C_T_ method as described in the Applied Biosystems (http://www.appliedbiosystems.com) user bulletin #2 for the ABI Prism 7700 sequence detection system based on all measurements of the respective genotypes/conditions.

Fat body tissue from 6-d-old (Figure 2B) to 13-d-old (Figure 3B) adults was prepared as follows for ex vivo confocal laser scanning microscopy. The abdomens of male flies were manually removed, opened, and the fat body tissue attached to the cuticle mechanically released into mounting medium (50% glycerol/PBS, Nile Red [Nile red stock 10% in DMSO] 1:55,000 [Molecular Probes/Invitrogen, http://www.invitrogen.com]). The tissue was analyzed within 2 h after mounting using a Leica TCS SP2 LSM (Leica, http://www.leica-microsystems.com) with 561-nm excitation/600–657-nm emission wavelength.

### Statistical analysis.

Mathematical significance of differences between datasets was analyzed using the unpaired *t* test and expressed as *p*-values.

## Supporting Information

Figure S1 Molecular Organization of the *AKHR* Gene Locus and *AKHR*-Dependent Storage-Fat Mobilization (A) Genomic organization (exons in white, coding exons in grey) of the *CG11188* and the *AKHR* gene locus on chromosome 2L at cytogenetic position 27A1. Localization of P element–integration A1332 in the fly stock *CG11188^A1332^*.
(B) Induced fat-storage reduction upon fat body–targeted *AKHR* expression via the A1332 P element or an independent UAS-*AKHR* transgene compared to controls. “Inducer +” refers to the presence of the fat body–specific *FB-Gal4* chromosome; “Inducer −” refers to the presence of the genetically matched control chromosome *FBJO* (for details, see complete fly stock genotypes in Table 1).
(453 KB EPS)

Figure S2 *AKHR* Function Is Not Essential for *brummer*-Induced Fat-Storage Mobilization Organismal fat storage is reduced upon induction of a transgene causing *bmm* overexpression in the fat body of control (compare *bmm* induced vs. *bmm* uninduced) as well as *AKHR^1^* mutant flies (compare *AKHR^1^ bmm* induced vs. *AKHR^1^ bmm* uninduced). “Induced” refers to the presence of the fat body–specific *FB-Gal4* chromosome, “uninduced” to the presence of the genetically matched control chromosome *FBJO* (for details, see complete fly stock genotypes in Table 1).
(363 KB EPS)

Figure S3 Organismal Lipid Composition of Fat *AKHR* and *brummer* Single Mutants Compared to Extremely Obese *AKHR brummer* Double-Mutant Flies Body fat accumulation in *AKHR* and *bmm* single mutants *(AKHR^1^* and *bmm^1^)* compared to controls *(AKHR^rev^ bmm^rev^)* is due to triacylglycerol (TAG) increase. Increased TAG content and an uncharacterized glyceride species (TAGX) add up to the extreme fat accumulation in *AKHR bmm* double-mutant flies. Note that DAG does not substantially contribute to the observed genotype-specific changes in the total glyceride content, although there is a significant increase (in *bmm* mutants) and decrease (in *AKHR bmm* double mutants) of DAG compared to the control.
(368 KB EPS)

Figure S4 *AKHR* and *brummer* Functions Are Not Essential for Starvation-Induced Glycogen Mobilization Complete depletion of glycogen stores during starvation of *AKHR* and *bmm* single mutants *(AKHR^1^* and *bmm^1^),* as well as of *AKHR bmm* double mutants (*AKHR^1^ bmm^1^*), is similar to the genetically matched control *(AKHR^rev^)*. Note the reduced glycogen content in ad libitum–fed *AKHR^1^ bmm^1^* double-mutant flies.
(369 KB EPS)

Figure S5 Lipolytic Activity Analysis of Fat Body Cells from Fed or 6-h Starved Flies TAG (A) and DAG (B) lipolysis activity of fat body cell lysate fractions from immature adult control flies (genotype: *AKHR^rev^ bmm^rev^*). The majority of basal TAG and DAG lipolytic activity resides in the cytosolic fraction. Starvation-induced TAG lipolysis is detectable in the cytosolic and the lipid droplet fractions, whereas fat body cells show no inducible DAG lipolysis. There is no significant genotype-specific or starvation-induced difference in DAG (C) or cholesterol oleate (D) lipolysis in cells from flies lacking *AKHR, bmm,* or *AKHR* and *bmm* functions *(AKHR^1^, bmm^1^,* and *AKHR^1^ bmm^1^)* compared to control flies *(AKHR^rev^ bmm^rev^)*. Black bars, fed flies; grey bars, starved flies.
(413 KB EPS)

Protocol S1 Supporting Materials and Methods, and Supporting References (33 KB DOC)

### Accession Numbers

The FlyBase ((http://flybase.bio.indiana.edu) accession number for the *AKHR* (or *CG11325*) gene (FlyBase name: *Gonadotropin-releasing hormone receptor [GRHR])* is FBgn0025595.

## Abbreviations

*Adh*: *Alcohol dehydrogenase*
AKH: adipokinetic hormone
AKHR: adipokinetic hormone receptor
ATGL: adipose triglyceride lipase
CGI-58: comparative gene identification 58
DAG: diacylglycerol
Hsl: hormone-sensitive lipase
LSD: lipid storage droplet
PKA: protein kinase A
TAG: triacylglycerol
UAS: upstream activation sequence
