# Supplementary material for: Dual Lipolytic Control of Body Fat Storage and Mobilization in Drosophila
Source: PLoS Biol. 2007 May 8;5(6):e137. doi: 10.1371/journal.pbio.0050137 (PMC1865564; doi:10.1371/journal.pbio.0050137)
Supplement: Supplementary file 1 [file pbio.0050137.sd001.doc]

## Supporting materials and methods

***Lipolysis assays and neutral lipid class quantification***

DAG cleavage of 3-10µl of total lysates, LD, membranes and cytosol was performed as described [1] with some modifications. Briefly, 60µmoles of 1(3)-[^3^H]oleoyl-2-*O*-oleylglycerol (2 µCi/µmol; Biotrend Cologne) were mixed with 1.8mg of phosphatidylcholine (soy bean) in chloroform. After careful evaporation and subsequent addition of 1.2ml of 20mM Tris/HCl (pH 7.4), 150mM NaCl, the mixture was sonicated (14°C, 2min, setting 2, Branson sonifier, type B12). After cooling to 4°C, 0.3ml of 0.5g/ml BSA in the same buffer was added and the sonication was repeated (4°C, 2min). For the assay, 30µl of substrate solution was mixed with 5µl of 5mM EDTA, 5µl of 5mM DTT and up to 10µl of sample and adjusted to a final volume of 50µl with buffer A and then incubated (2h, 20°C, 100 cycles/min). The reaction was terminated and the released radioactively labelled oleate was recovered by a one-step liquid-liquid partition system by addition of 800µl of methanol/chloroform/heptane (1.41/1.25/1, by vol.) and 250µl of 0.1M potassium carbonate/0.1M boric buffer (pH10.5). The mix was shaken vigorously for 20sec and then centrifuged (800x*g*, 20min). The upper phase (250µl) was used for scintillation counting.

Cholesterol oleate cleavage of 3-10µl of total lysates, LD, membranes and cytosol was measured as described previously [2,3] with some modifications. Briefly, for 2.5ml of substrate solution, 1.8mM cholesteryl[^3^H]oleate (200µCi, Amersham) was emulsified with 5.6mg of phosphatidylcholine/phosphatidylinositol, (3/1 by vol.) in chloroform. After evaporation and addition of 2ml of 0.1M potassium phosphate (pH 7.0) and heating to 37°C, the substrate suspension was sonicated (2x1 min with 1 min interval, see above). After the second sonication, 0.5ml of 0.5g/ml BSA in the same buffer was added. The lipase assay reaction was carried out as described for the DAG lipase assay (see above) with10 µl of sample.

Lipolytic activity was calculated as dpm±SD of fatty acid released per 1 h and 1µg of protein or 1µl of sample volume. Protein was determined using the Pierce BCA assay kit (Perbio Science) with BSA as standard. Lipase activities and protein were determined in three independent measurements.

For determination of neutral lipid species 20 flies were homogenized in 400µl of PBS (10 strokes with a tight-fitting teflon-in-glass homogenizer followed by 3x10sec ultrasonic treatment). The homogenate was supplemented with 400µl chloroform/methanol (2/1, by vol.) containing 0.1% (w/v) butylated hydroxytoluene [4] and incubated (1h, 30°C). After phase separation, 400µl of the upper aqueous phase was removed, dried (SpeedVac) and then suspended in 40µl of PBS containing 0.1% SDS for protein determination. About two thirds of the lower organic phase (300µl) were removed and along with some insoluble materials filtered through a medium-porosity sintered glass funnel. The filtered extract was evaporated to dryness (SpeedVac) and stored under N_2_ at –20°C in 20µl tetrahydrofurane. Neutral lipids were resolved by two-dimensional TLC of 5µl samples on HPTLC plates (Silica Gel 60, Merck) in benzene/hexane (10/1 by vol.) for the separation of triacylglyceride and cholesterol esters (1^st^ dimension), and in hexane/diethyl ether/acetic acid (50/50/1 by vol.) for the separation of diacylglycerides, monoacylglycerides, free fatty acids and cholesterol (2^nd^ dimension). Lipid standards (Avanti Polar Lipids) were run in parallel on the same plates in the same chamber. For TLC determination of the amount of the various lipid classes according to White et al. [5], the dried plates were sprayed with a 0.05% solution of primulin dye (Sigma; 1:100 dilution of a 5% aqueous stock into acetone:water, 8:2 by vol.). The plates were sprayed in a uniform manner until visibly moist, then dried for 2min and finally scanned by laser-excited fluorescent detection (Molecular Dynamics, Storm 860 imaging system). Spots were quantified by integration of variable pixel intensities on Imagequant software and compared to standard curves obtained with increasing volumes of triolein and diolein.

For characterization of neutral lipid species, spot materials comigrating with the triolein and diolein standards (triacylglyceride, diacylgyceride) as well as of materials migrating with R_f_-values between those of the standards (TAGX) were scraped from the plates into glass centrifuge tubes. The silica was extracted with 4x2ml of warm chloroform, resulting in a recovery of 85-90% of known amounts of triolein. The chloroform phases were pooled and dried under a stream of N_2_. Portions were dissolved in isopropanol and then quantitatively evaluated for glycerol and fatty acids (Cobas Bio automatic analyzer, Roche Diagnostics).

For size determination of neutral lipid species, other portions of the dried samples were dissolved in 100µl of ethyl acetate (Merck, Germany) and three 2.5µl aliquots at 30 seconds intervals, each, analyzed by gas-liquid chromatography as previously described [6], using an Ultra 1 Hewlett-Packard fused silica capillary column (0.5m x 0.31mm i.d.) coated with crosslinked dimethyl siloxane and 0.086bar N_2_ as carrier gas. Oven temperature was programmed from 205°C to 345°C at a rate of 6°C/min and the carrier gas was hydrogen (0.5bar). Lipid classes were identified by a mixture of various tri- and diacylglyceride standards (Sigma or Avanti Polar Lipids). 3µg sn-1,3-dimyristoyl glycerol and 1µg triheptadecanoyl glycerol prevented from oxidation by 8µg of butylated hydroxytoluene were coinjected with each sample as internal references. Ethyl acetate and neutral lipid eluates were trapped in pyridine at 20°C. The recovery of neutral lipids was checked using the same column and the standards (di- and triacylglyceride 60-80% / 25-40%).

*AKHR^1^ bmm^1^* double mutant flies accumulate an unknown and apparently heterogenous lipid species (TAGX), which according to size determination by GLC, partially consists of triacylglyceride (C51, C55) presumably built up by unsaturated acyl chains. However, it cannot be excluded at present, that TAGX also contains diacylglyceride species (apparently not used as standards for GLC), which may explain the migration behavior during TLC (broad range of R_f_-values) and the intermediate fatty acid/glycerol ratio (2.69/1).

## Supporting references

1. Tornqvist H, Bjorgell P, Krabisch L, Belfrage P (1978) Monoacylmonoalkylglycerol as a substrate for diacylglycerol hydrolase activity in adipose tissue. J Lipid Res 19: 654-656.

2. Ali YB, Carriere F, Verger R, Petry S, Müller G, et al. (2005) Continuous monitoring of cholesterol oleate hydrolysis by hormone-sensitive lipase and other cholesterol esterases. J Lipid Res 46: 994-1000.

3. Fredrikson G, Stralfors P, Nilsson NO, Belfrage P (1981) Hormone-sensitive lipase of rat adipose tissue. Purification and some properties. J Biol Chem 256: 6311-6320.

4. Folch J, Lees M, Sloane Stanley GH (1957) A simple method for the isolation and purification of total lipides from animal tissues. J Biol Chem 226: 497-509.

5. White T, Bursten S, Federighi D, Lewis RA, Nudelman E (1998) High-resolution separation and quantification of neutral lipid and phospholipid species in mammalian cells and sera by multi-one-dimensional thin-layer chromatography. Anal Biochem 258: 109-117.

6. Barrans A, Collet X, Barbaras R, Jaspard J, Manent C, Vieu C, Chap H, Perret B

(1994) Hepatic lipase induces the formation of pre-beta 1 high density lipoprotein (HDL) from triacylglycerol-rich HDL2. A study comparing liver perfusion to in vitro incubation with lipases. J Biol Chem 269: 11572-11577.
